# Nickle-cobalt alloy nanocrystals inhibit activation of inflammasomes

**DOI:** 10.1093/nsr/nwad179

**Published:** 2023-06-26

**Authors:** Jun Lin, Liang Dong, Yi-Ming Liu, Yi Hu, Chen Jiang, Ke Liu, Liu Liu, Yong-Hong Song, Mei Sun, Xing-Cheng Xiang, Kun Qu, Yang Lu, Long-Ping Wen, Shu-Hong Yu

**Affiliations:** Department of Neurosurgery, The First Affiliated Hospital of USTC, School of Basic Medical Sciences, Division of Molecular Medicine, Department of Chemistry, Institute of Biomimetic Materials & Chemistry, Division of Nanomaterials & Chemistry, Hefei National Research Center for Physical Sciences at the Microscale, University of Science and Technology of China, Hefei 230027, China; Department of Neurosurgery, The First Affiliated Hospital of USTC, School of Basic Medical Sciences, Division of Molecular Medicine, Department of Chemistry, Institute of Biomimetic Materials & Chemistry, Division of Nanomaterials & Chemistry, Hefei National Research Center for Physical Sciences at the Microscale, University of Science and Technology of China, Hefei 230027, China; The Cancer Hospital of the University of Chinese Academy of Sciences (Zhejiang Cancer Hospital), Institute of Basic Medicine and Cancer (IBMC), Chinese Academy of Sciences, Hangzhou 310022, China; Department of Neurosurgery, The First Affiliated Hospital of USTC, School of Basic Medical Sciences, Division of Molecular Medicine, Department of Chemistry, Institute of Biomimetic Materials & Chemistry, Division of Nanomaterials & Chemistry, Hefei National Research Center for Physical Sciences at the Microscale, University of Science and Technology of China, Hefei 230027, China; Department of Neurosurgery, The First Affiliated Hospital of USTC, School of Basic Medical Sciences, Division of Molecular Medicine, Department of Chemistry, Institute of Biomimetic Materials & Chemistry, Division of Nanomaterials & Chemistry, Hefei National Research Center for Physical Sciences at the Microscale, University of Science and Technology of China, Hefei 230027, China; Department of Neurosurgery, The First Affiliated Hospital of USTC, School of Basic Medical Sciences, Division of Molecular Medicine, Department of Chemistry, Institute of Biomimetic Materials & Chemistry, Division of Nanomaterials & Chemistry, Hefei National Research Center for Physical Sciences at the Microscale, University of Science and Technology of China, Hefei 230027, China; Department of Neurosurgery, The First Affiliated Hospital of USTC, School of Basic Medical Sciences, Division of Molecular Medicine, Department of Chemistry, Institute of Biomimetic Materials & Chemistry, Division of Nanomaterials & Chemistry, Hefei National Research Center for Physical Sciences at the Microscale, University of Science and Technology of China, Hefei 230027, China; Department of Neurosurgery, The First Affiliated Hospital of USTC, School of Basic Medical Sciences, Division of Molecular Medicine, Department of Chemistry, Institute of Biomimetic Materials & Chemistry, Division of Nanomaterials & Chemistry, Hefei National Research Center for Physical Sciences at the Microscale, University of Science and Technology of China, Hefei 230027, China; Anhui Province Key Laboratory of Advanced Catalytic Materials and Reaction Engineering, School of Chemistry and Chemical Engineering, Hefei University of Technology, Hefei 230009, China; Department of Neurosurgery, The First Affiliated Hospital of USTC, School of Basic Medical Sciences, Division of Molecular Medicine, Department of Chemistry, Institute of Biomimetic Materials & Chemistry, Division of Nanomaterials & Chemistry, Hefei National Research Center for Physical Sciences at the Microscale, University of Science and Technology of China, Hefei 230027, China; The WUT-AMU Franco-Chinese Institute, Wuhan University of Technology, Wuhan 430070, China; Department of Neurosurgery, The First Affiliated Hospital of USTC, School of Basic Medical Sciences, Division of Molecular Medicine, Department of Chemistry, Institute of Biomimetic Materials & Chemistry, Division of Nanomaterials & Chemistry, Hefei National Research Center for Physical Sciences at the Microscale, University of Science and Technology of China, Hefei 230027, China; Institute of Artificial Intelligence, Hefei Comprehensive National Science Center, Hefei 230027, China; Anhui Province Key Laboratory of Advanced Catalytic Materials and Reaction Engineering, School of Chemistry and Chemical Engineering, Hefei University of Technology, Hefei 230009, China; Department of Neurosurgery, The First Affiliated Hospital of USTC, School of Basic Medical Sciences, Division of Molecular Medicine, Department of Chemistry, Institute of Biomimetic Materials & Chemistry, Division of Nanomaterials & Chemistry, Hefei National Research Center for Physical Sciences at the Microscale, University of Science and Technology of China, Hefei 230027, China; Department of Neurosurgery, The First Affiliated Hospital of USTC, School of Basic Medical Sciences, Division of Molecular Medicine, Department of Chemistry, Institute of Biomimetic Materials & Chemistry, Division of Nanomaterials & Chemistry, Hefei National Research Center for Physical Sciences at the Microscale, University of Science and Technology of China, Hefei 230027, China

**Keywords:** inflammasome, nickel-cobalt nanoparticle, *Neat1*, colitis, NLRP3

## Abstract

Activation of inflammasomes—immune system receptor sensor complexes that selectively activate inflammatory responses—has been associated with diverse human diseases, and many nanomedicine studies have reported that structurally and chemically diverse inorganic nanomaterials cause excessive inflammasome activation. Here, in stark contrast to reports of other inorganic nanomaterials, we find that nickel-cobalt alloy magnetic nanocrystals (NiCo NCs) actually inhibit activation of NLRP3, NLRC4 and AIM2 inflammasomes. We show that NiCo NCs disrupt the canonical inflammasome ASC speck formation process by downregulating the lncRNA *Neat1*, and experimentally confirm that the entry of NiCo NCs into cells is required for the observed inhibition of inflammasome activation. Furthermore, we find that NiCo NCs inhibit neutrophil recruitment in an acute peritonitis mouse model and relieve symptoms in a colitis mouse model, again by inhibiting inflammasome activation. Beyond demonstrating a highly surprising and apparently therapeutic impact for an inorganic nanomaterial on inflammatory responses, our work suggests that nickel- and cobalt-containing nanomaterials may offer an opportunity to design anti-inflammatory nanomedicines for the therapeutics of macrophage-mediated diseases.

## Teaser

Nickel-Cobalt nanocrystals inhibited NLRP3, NLRC4, and AIM2 inflammasomes activation by downregulating the transcription of lncRNA Neat1, and significantly relieved symptoms of acute peritonitis and colitis mouse model.

## INTRODUCTION

Inflammasome activation is essential for innate immune responses as it facilitates the clearance of pathogens and/or damaged cells [1,2]. Recently, it has been revealed that the activation of inflammasomes also participates in adaptive immunity [3,4], and some nanomaterials can activate inflammasomes to serve as an adjuvant vaccine with increased immunogenicity [5–8]. However, inflammasome activation can also be a major driver of autoimmune and metabolic disorders, including Alzheimer's disease, Parkinson's disease, pulmonary fibrosis, inflammatory bowel disease, type II diabetes, gout, ovarian aging and atherosclerosis [1,2,9,10]. A large number of stimuli, including UV radiation [11], viral and bacterial infection [12–14], extracellular adenosine triphosphate (ATP) [15,16] and β-amyloid plaques [17], as well as particles such as monosodium urate crystals (MSU) [18], alum [7], cholesterol [19] and asbestos [20], are known to activate NLRP3 inflammasomes. Work to date has indicated that most examined nanomaterials, including silicon oxide [6,20,21], metal and metal-based nanomaterials [5,8,22–24] and carbon nanomaterials [25], etc., can activate inflammasomes (mainly NLRP3 inflammasomes), by causing K^+^ efflux [5], Ca^2+^ influx [24], ROS generation [20], lysosome destruction [21,24] or disruption of autophagy [8]. In response to specific stimuli, the relevant inflammasome sensors (e.g. nucleotide-binding domains and leucine-rich repeat receptors (NLRs) or absent in melanoma 2 (AIM2)-like receptors (ALRs)) assemble with an adapter protein (an apoptosis-associated speck-like protein containing a CARD, denoted as ASC) and pro-caspase-1 to form inflammasomes, resulting in the cleavage and activation of caspase-1. Subsequently, activated caspase-1 can cleave proinflammatory IL-1 family cytokines into their bioactive forms, IL-1β and IL-18 [9,26].

Due to the relationship of inflammasome activation with autoimmune and metabolic disorders, effective inflammasome inhibitors are highly required. So far, a series of small molecules, including MCC-950 [27], oridonin [28], omega-3 fatty acids [29] and fenamate non-steroidal anti-inflammatory drugs [30], have been identified to inhibit the activation of inflammasomes. Macrophages are the main cell type that produces inflammasomes, and are also the first and primary cell type in the body that processes nanoparticles [31,32]. This apparent macrophage targeting has motivated much research in nanomedicine, suggesting the possibility that nanoparticles may outperform small molecule inhibitors based on their processing by macrophages, enabling precise and highly efficient delivery of therapeutic agents. Very recently, a cationic lipid-assisted pegylated-poly(lactic-co-glycolic acid) nanoparticle (CLAN) was shown to successfully deliver Cas9 mRNA (mCas9) and guide RNA (gRNA) into macrophages, which achieved targeted editing of the NLRP3 gene to inhibit inflammasome activation [33]. However, looking beyond examples of loading a nanomaterial with inflammasome inhibitors notwithstanding [34,35], we are unaware of any reports showing that a nanoparticle *per se* can inhibit inflammasome activation. That is, while we know that a variety of nanoparticles cause inflammasome activation, little if any work has explored the idea that some useful property inherent to a particular type of nanoparticle could actually be exploited to inhibit inflammasome activation.

**Figure 1.**
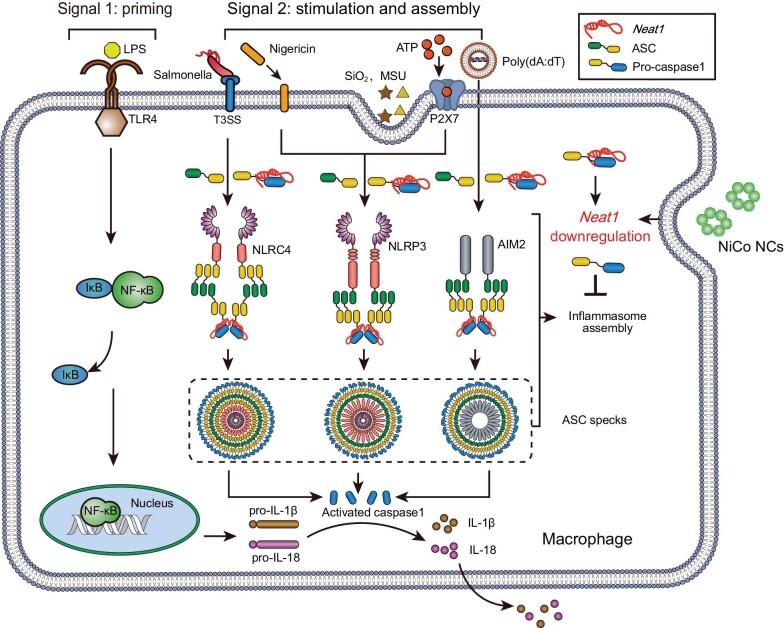
Schematic illustration for inhibition of NLRP3, NLRP4 and AIM2 inflammasome activation by NiCo NCs. NiCo NCs inhibited activations of inflammasomes induced by different agonists. We used RNA sequencing to search for the mechanism by which NiCo NCs broadly inhibit inflammasomes activation. *Neat1*, a long noncoding RNA that has been reported to bind to NLRP3, NLRC4 and AIM2 inflammasomes to enhance their assembly, was found to be significantly downregulated after NiCo NCs treatment.

Here we found that nickel-cobalt alloy nanocrystals (NiCo NCs) efficiently inhibit the activation of NLRP3 inflammasomes by multiple agonists. NiCo NCs were also confirmed to inhibit the activation of NLRC4 and AIM2 inflammasomes, indicating their broad-spectrum inhibitory effects against diverse canonical inflammasome types (Fig. 1). According to the ASC speck formation assay result, NiCo NCs disrupted the assembly of inflammasomes to inhibit these three kinds of inflammasome activation. RNA sequencing analysis revealed that NiCo NCs inhibited the formation of ASC speck by downregulating of a long non-coding RNA *Neat1*. Furthermore, NiCo NCs were observed to relieve symptoms of acute peritonitis and colitis in mice, which was attributed to its inherent inhibition of inflammasome activation.

## RESULTS AND DISCUSSION

### Inhibition of NLRP3 inflammasome activation

**Figure 2.**
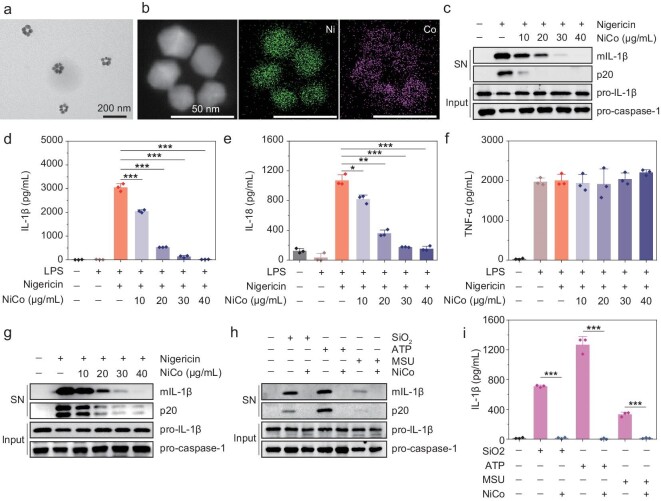
NiCo NCs inhibit the nigericin induced NLRP3 inflammasomes activation. (a) TEM image of NiCo NCs. (b) EDS mapping of NiCo NCs showing element distribution of Ni and Co. (c and d) LPS-primed BMDMs were treated with different doses of NiCo NCs and then stimulated with nigericin. Medium supernatant (SN) and cell lysis (Input) were analyzed by immunoblotting for mature IL-1β (mIL-1β), activated caspase-1 (p20), pro-IL-1β and pro-caspase-1. (d–f) Supernatants were also analyzed by ELISA for (d) IL-1β, (e) IL-18 and (f) TNF-α release. (g) PMA-differentiated THP-1 cells were treated with different doses of NiCo NCs and then stimulated with nigericin. Medium supernatant (SN) and cell lysis (Input) were analyzed by immunoblotting. (h and i) LPS-primed BMDMs were treated with 30 μg/mL NiCo NCs and then stimulated with SiO_2_, ATP or MSU. Medium supernatant (SN) and cell lysis (Input) were analyzed by immunoblotting as indicated in (h), supernatants were analyzed by ELISA for IL-1β release (i). All data are presented as mean ± s.d., *n * =  3 independent experiments. Statistical significance was assessed using one-way analysis of variance (ANOVA). *P < 0.05, **P < 0.01, ***P < 0.001.

As the main cells which express inflammasome genes, macrophages function as major endocytic cells, which are also the first and primary cell type in the body that processes nanoparticles. This motivated our effort to examine whether inflammasome activation can be appreciably inhibited by exposure to certain nanomaterials, and we finally found such a nanomaterial. NiCo NCs were prepared via a previously reported microwave irradiation method using a 7 : 3 molar ratio of Ni : Co precursors (Supplementary Fig. 1) [36]. We successfully obtained NiCo NCs with a diameter of 20–30 nm, and confirmed their expected ring-like structure, which forms on account of a magnetic dipolar interaction (Fig. 2a and b, and Supplementary Fig. 2). The zeta potential of NiCo NCs (−26.9 ± 1.23 mV) was measured using zeta seizer. Moreover, the results of the zeta potential for the NiCo NCs suspension showed that proteins and organic molecules in cell culture medium have minimal impact on the interactions between NiCo NCs and the cells (Supplementary Fig. 3) [37].

Numerous studies have reported that diverse nanomaterials induce NLRP3 inflammasome activation; such studies have typically used caspase-1 and IL-1β as activation markers [25]. We found that NiCo NCs pretreatment attenuated both caspase-1 activation and IL-1β maturation in LPS-primed bone-marrow-derived macrophages (BMDMs) induced by nigericin (a commonly used NLRP3 inflammasome agonist), and the attenuation is NiCo NCs dose-dependent (Fig. 2c and d). And at a concentration of 30 μg/mL, the release of IL-1β could be almost completely inhibited by NiCo NCs. Further, the expression of pro–IL-1β was not attenuated upon NiCo NCs treatment, suggesting that NiCo NCs do not interfere with the LPS-induced priming process in BMDMs (Fig. 2c). Similarly, we found that NiCo NCs pretreatment also blocked nigericin-induced secretion of IL-18, another inflammasome-dependent cytokine, again in a dose-dependent manner (Fig. 2e). Analysis of TNF-α production—an inflammasome-independent cytokine—was not affected by NiCo NCs treatment, a finding which suggested that NiCo NCs may inhibit IL-1β production through suppressing inflammasome activation (Fig. 2f). The release of IL-1β was also significantly inhibited even when NiCo NCs were added after the activation of the NLRP3 inflammasome (Supplementary Fig. 4). In addition, 30 μg/mL NiCo NCs treatment had no effect on cell viability of BMDMs (Supplementary Fig. 5). Extending these insights beyond a single cell type, we again observed that NiCo NCs treatment resulted in dose-dependent inhibition of caspase-1 activation and IL-1β maturation in a human leukemic cell line (THP-1) (Fig. 2g).

To exclude the possibility of some nigericin-specific effect in the observed inhibition of NLRP3 inflammasome activation, we also conducted experiments which incorporated multiple well-established NLRP3 agonists as control groups, including SiO_2_ NPs, ATP and monosodium urate (MSU) [21,29]. Supporting the generality of the observed effects, NiCo NCs inhibited caspase-1 activation and IL-1β maturation as induced by SiO_2_, by ATP and by MSU treatment (Fig. 2h and i). These results indicated that NiCo NCs inhibit the activation of NLRP3 inflammasomes caused by multiple agonists.

### Inhibition of NLRC4 and AIM2 inflammasomes activation

**Figure 3.**
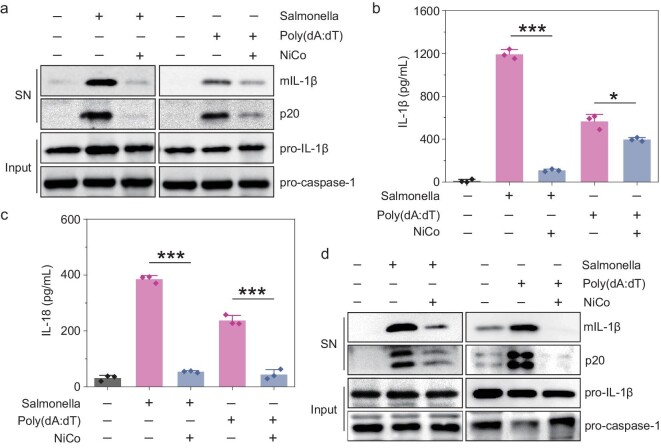
NiCo NCs can inhibit the activation of NLRC4 and AIM2 inflammasomes. (a–c) LPS-primed BMDMs were pretreated with 30 μg/mL of NiCo NCs and then stimulated with salmonella or poly (dA : dT). Medium supernatant (SN) and cell lysis (Input) were analyzed by immunoblotting. Supernatants were also analyzed by ELISA for the release of (b) IL-1β and (c) IL-18. (d) PMA-differentiated THP-1 cells were pretreated with 30 μg/mL of NiCo NCs and then stimulated with salmonella or poly (dA : dT). Medium supernatant (SN) and cell lysis (Input) were analyzed by immunoblotting. All data are presented as mean ± s.d., *n*  =  3 independent experiments. Statistical significance was assessed using one-way analysis of variance (ANOVA). *P < 0.05, ***P < 0.001.

We also tested if NiCo NCs may exert inhibitory effects against the activation of other canonical inflammasomes, including the extensively studied NLRC4 and AIM2 inflammasomes. Indeed, similar to our observations for inhibition of NLRP3 inflammasome activation, we found that NiCo NCs blocked caspase-1 activation and IL-1β maturation in LPS-primed BMDMs stimulated with salmonella (an agonist of NLRC4 inflammasomes) or with poly (dA : dT) (an agonist of AIM2 inflammasomes) (Fig. 3a and b). As shown in Fig. 3c, the secretion of IL-18 induced by salmonella or poly (dA : dT) was also dramatically attenuated by NiCo NCs treatment. We observed that NiCo NCs treatment caused inhibition of caspase-1 activation and IL-1β maturation in THP-1 cells which were activated with salmonella or poly (dA : dT) (Fig. 3d). Together, these results underscore that NiCo NCs exert broad-spectrum inhibitory effects on the activation of NLRP3, NLRC4 and AIM2 inflammasomes.

### Mechanism of inhibition on the activation of inflammasomes via NiCo NCs

**Figure 4.**
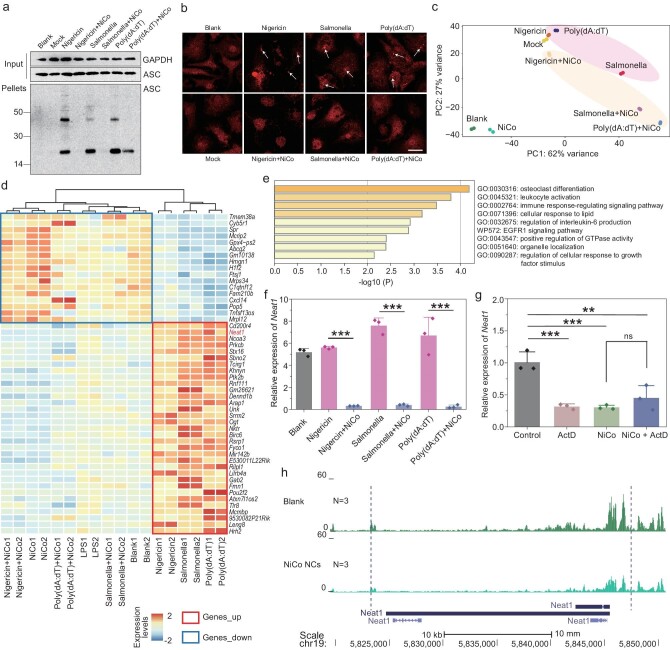
Mechanism of inhibition on the activation of inflammasome via NiCo NCs. (a) LPS-primed BMDMs were pretreated with 30 μg/mL of NiCo NCs and then stimulated with nigericin, salmonella or poly (dA : dT). Cell lysis (Input) and cross-linked cytosolic pellets were analyzed by immunoblotting. Blank = BMDMs without LPS-priming, Mock = LPS-primed BMDMs without stimulation. (b) Anti-ASC immunofluorescence analysis of LPS-primed BMDMs were treated as in (a). The white arrow indicates example of ASC specks. (c) Principal component analysis (PCA) was performed based on gene expression from the 18 samples in 9 conditions. Each dot is a sample, the samples in the figure are colour-coded by different treatment. (d) Heatmap showing normalized expression levels of two specific gene sets (Genes_up and Genes_down) across 18 samples of 9 conditions. Genes are sorted from top to bottom according to the mean foldchange values obtained by DEG analysis of three groups (Nigericin vs Nigericin + NiCo; Salmonella vs Salmonella + NiCo; Poly(dA : dT) vs Poly(dA : dT) + NiCo). Genes_up : genes that are upregulated during inflammasome activation and are downregulated after NiCo NCs co-treatment; Genes_down : genes that are downregulated during inflammasome activation and are upregulated after NiCo NCs co-treatment. (e) Pathway enrichment analysis of the ‘Genes_up’ listed in revised Fig. 4d. (f) Real-time RT-PCR analysis of the relative expression of *Neat1* in different treated groups. (g) Real-time RT-PCR analysis of the relative expression of *Neat1* after NiCo NCs treatment in the presence or absence of actinomycin D (ActD). (h) Normalized ATAC-seq signal profiles at the gene body and promoter region of *Neat1* (upstream 2000 bp and downstream 2000 bp). *N* represents the number of biological replicates. All data are presented as mean ± s.d., *n*  =  3 independent experiments. Statistical significance was assessed using one-way analysis of variance (ANOVA). **P < 0.01, ***P < 0.001, ns = not significant or *P* > 0.05.

To reveal the mechanism by which NiCo NCs inhibit the activation of NLRP3, NLRC4 and AIM2 inflammasomes, we first assessed the expression of ASC, which is the common protein present in all three of these inflammasome types. As shown in Fig. 4a, the expression of ASC was not affected by NiCo NCs treatment. We also examined formation of ASC oligomers, a key event in NLRP3, NLRC4 and AIM2 inflammasome activation [27,38]. Specifically, cytosolic fractions from BMDM lysates were cross-linked, and ASC monomers and higher order complexes were observed after stimulation with nigericin, salmonella, or poly (dA : dT), and the ASC complex formation was significantly attenuated by NiCo NCs treatment (Fig. 4a). Moreover, the inhibitory effect of NiCo NCs on ASC speck formation was also identified by immunofluorescence assays : ASC was evenly distributed in the untreated or LPS-primed cells, but upon nigericin, salmonella, or poly (dA : dT) activation, ASC appeared as bright specks under fluorescence microscopy, and NiCo NCs treatment clearly attenuated the ASC speck formation process (Fig. 4b). These results suggest that NiCo NCs can inhibit activation of NLRP3, NLRC4 and AIM2 inflammasomes by decreasing the formation of ASC specks.

To further explore the mechanism that NiCo NCs inhibited the formation of ASC speck, RNA sequencing, a method that is considered the most powerful and robust technique for measuring gene expression at genome-wide level, was carried out on different treated BMDM groups to find genes that may be involved in this process. The Pearson correlation coefficient of RNA sequencing signals suggested excellent reproducibility between the biological replicates (Supplementary Fig. 6). Despite the KEGG pathway enrichment analysis of differential expressed genes (DEGs) showed an enrichment in pathways such as TNF signaling pathway, rheumatoid arthritis, and MAPK signaling pathway following treatment with NiCo NCs compared to the Blank group (Supplementary Fig. 7), the principal component analysis (PCA) performed on the gene expression of all samples indicated that the NiCo NCs group and Blank group exhibited greater similarity in gene expression (Fig. 4c). And samples treated with inflammasome agonists and samples co-treated with inflammasome agonists and NiCo NCs can be seperated into two clusters (Fig. 4c). We then performed a DEGs analysis to find the genes that caused the above two groups to be differentiated. Figure 4d showed the genes that upregulated/downregulated significantly after nigericin, salmonella, or poly (dA : dT) treatment compared with the LPS-priming group (Mock), and their expression downregulated/upregulated significantly after NiCo NCs co-treatment, we named these two gene lists ‘Genes_up’ and ‘Genes_down,’ respectively. Supplementary Figs 8 and 9 show how we obtained the list by comparing different groups.

The pathway enrichment analysis showed that the Genes_up were enriched in immune respone-related functions, including osteoclast differentiation, leukocyte activation, immune response-regulating signaling pathway (Fig. 4e). These findings suggest that these genes are potential candidates for suppressing inflammation induced by NiCo NCs. Notably, *Neat1*, which was upregulated by three inflammasome agonists treatment and downregulated by inflammasome agonists and NiCo NCs co-treatment, is a reported long noncoding RNA (lncRNA) that plays an important role in inflammasome activation. *Neat1* was reported to associate with the NLRP3, NLRC4 and AIM2 inflammasomes to enhance their assembly and subsequent pro–caspase-1 processing. *Neat1* deficiency significantly inhibits the activation of NLRP3, NLRC4 and AIM2 inflammasomes in BMDMs [39]. We further verified that NiCo NCs treatment can significantly downregulate *Neat1* by real-time reverse transcription PCR (real-time RT-PCR) analysis (Fig. 4f).

The decrease of *Neat1* could result from either depressed transcription or enhanced RNA degradation. To determine which was the case for NiCo NCs, we measured the amount of *Neat1* in the presence and absence of actinomycin D (ActD), an unspecific transcription blocker. ActD or NiCo NCs treatment alone for 1.5 h significantly decreased the relative amount of *Neat1* compared to the control group, but NiCo NCs did not further decrease the amount of *Neat1* in the cells treated with ActD (Fig. 4g), which suggested that NiCo NCs treatment suppressed the transcription of *Neat1* rather than enhanced the degradation process. To shed more light on this, we performed Assay for Transposase-Accessible Chromatin with high-throughput sequencing (ATAC-seq), a method for mapping genome-wide chromatin accessibility [40,41]. The transcription start site (TSS) enrichment indicated the high quality of the dataset (Supplementary Fig. 10). We found the gene body and promoter region of *Neat1* were significantly less accessible in the NiCo NCs treated group compare to the Blank group (Fig. 4h), which further confirmed NiCo NCs treatment decreased the transcription of *Neat1*. Collectively, these results indicated that NiCo NCs inhibit the formation of ASC specks by downregulating the transcription of *Neat1*, thereby inhibiting the activation of NLRP3, NLRC4 and AIM2 inflammasomes.

### The necessity of NiCo NCs entering cells before exerting anti-inflammatory effects

**Figure 5.**
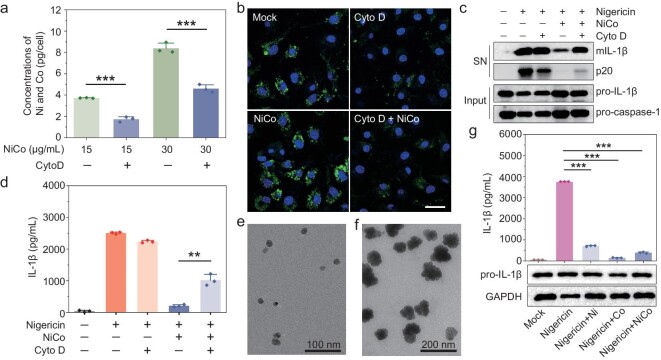
NiCo NCs must enter cells prior to exerting their anti-inflammatory effects. (a) ICP-MS assay for the concentration of Ni^2+^ and Co^2+^ in the NiCo NCs or NiCo NCs + Cyto D treated LPS-primed BMDMs. (b) The representative fluorescent pictures of LPS-primed BMDMs which were treated with 30 μg/mL NiCo NCs, 10 μM Cyto D or NiCo NCs + Cyto D for 2 h in the presence of 0.5 mg/mL FITC-dextran. Mock = LPS-primed BMDMs without stimulation. The nucleus was stained by 1 μg/mL Hoechst 33342. Scale bar = 20 μm. (c and d) LPS-primed BMDMs were treated with 30 μg/mL NiCo NCs, Cyto D or NiCo NCs + Cyto D for 2 h, and then stimulated with nigericin. Medium supernatant (SN) and cell lysis (Input) were analyzed by (c) immunoblotting and (d) supernatants were also analyzed by ELISA for IL-1β release. (e and f) TEM images of (e) Ni NPs and (f) Co NPs. (g) LPS-primed BMDMs were pretreated with 30 μg/mL of NiCo NCs, Ni NPs or Co NPs and then stimulated with nigericin. Cell lysis was analyzed by immunoblotting for pro-IL-1β and GAPDH. Supernatants were analyzed by ELISA for the release of IL-1β. All data are presented as mean ± s.d., *n*  =  3 independent experiments. **P < 0.01, ***P < 0.001.

Inductively coupled plasma mass spectrometry (ICP-MS) analysis revealed that different macrophage cell lines internalized NiCo NCs in a dose-dependent manner (Supplementary Fig. 11). To confirm that NiCo NCs enter cells prior to exerting anti-inflammatory effects, we conducted experiments with the widely used endocytosis inhibitor cytochalasin D (Cyto D), which is known to block the endocytosis of nanoparticles in BMDMs [24]. Cyto D treatment significantly reduced the extent of endocytosis in different concentrations of NiCo NCs-treated cells (Fig. 5a). Additionally, we used the fluorescein isothiocyanate (FITC) labelled-dextran (70 KD)—a known fluid-phase endocytosis indicator—to directly visualize the inhibitory effect of Cyto D treatment on endocytosis [42]. FITC-dextran cellular entry was obviously detected in LPS-primed and NiCo NCs-treated BMDMs, and this entry was abolished upon treatment with Cyto D (Fig. 5b). Importantly, we also observed in nigericin-stimulated cells that both caspase-1 activation and IL-1β maturation were suppressed by NiCo NCs treatment and confirmed that these suppression phenotypes could be recovered by inhibiting the endocytosis of NiCo NCs (Fig. 5c and d). Collectively, these results establish that NiCo NCs must enter cells prior to exerting their anti-inflammatory effects.

To figure out the suppression related to geometry or elements, we synthesized nickel nanoparticles (Ni NPs) and cobalt nanoparticles (Co NPs) in identical conditions as the control. Both Ni NPs and Co NPs have a morphology that is quite different from that of NiCo NCs (Fig. 5e and f). However, they were also able to significantly inhibit the activation of the NLRP3 inflammasomes (Fig. 5g). Therefore, we attribute the inhibitory effect of NiCo NCs to the presence of nickel and cobalt rather than the geometry. Those results suggested that nickel- and cobalt-containing nanomaterials may offer an opportunity to design nanomedicines with anti-inflammatory properties.

### Anti-inflammatory effects of NiCo NCs *in vivo*

**Figure 6.**
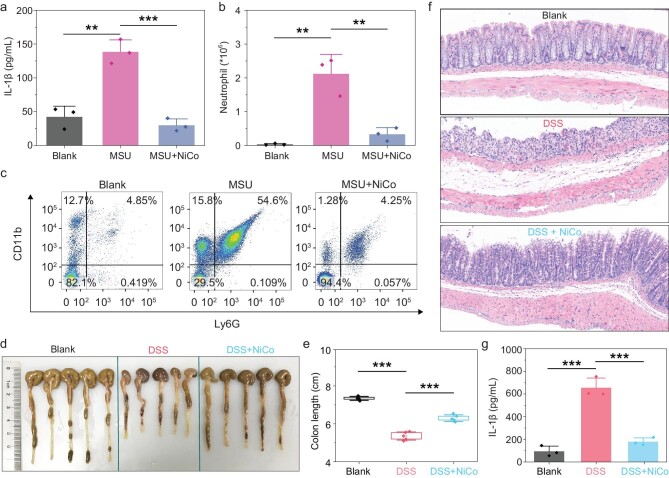
NiCo NCs suppressed NLRP3 inflammasome activation in acute peritonitis and colitis mouse models. (a) C57BL/6 mice were intraperitoneally injected with PBS (Blank), 3 mg MSU or 3 mg MSU + 300 μg NiCo NCs for 6 h. The supernatant of peritoneal perfusate was analyzed by ELISA for IL-1β release. (b and c) The recruitment of neutrophil into abdominal cavity of differently treated mice was analyzed by (b) flow cytometry and (c) is the representative picture. (d) Digital pictures and (e) statistical colon length of different groups of mice including DSS-free water (Blank), 2.5% DSS (red) and 2.5% DSS + 5 mg/kg NiCo NCs-fed (blue). C57BL/6 mice were sacrificed at day 8 and colon length was measured. (f) H&E staining of sections displayed severe disruption of the mucosal epithelium in DSS-treated mice and rescued by NiCo NCs co-treatment. (g) The colons from different treated mice were incubated in opti-MEM for 12 h and IL-1β releasing was tested by ELISA. All data are presented as mean ± s.d. Data in (a), (b) and (g) are from *n*  =  3 biologically independent samples. Data in (e) is from *n* = 5 biologically independent samples. Statistical significance was assessed using one-way analysis of variance (ANOVA). **P < 0.01, ***P < 0.001.

To verify the anti-inflammatory effects of NiCo NCs *in vivo*, we examined whether NiCo NCs can suppress MSU-induced peritoneal inflammation in a mouse model. For context, MSU deposition in joints is associated with development of gout and pseudogout via NLRP3 inflammasome activation [18], and MSU is widely used to induce peritoneal inflammation disease models in mice [28]. Upon MSU induction of the model, NiCo NCs treatment significantly reduced production of IL-1β in the abdominal cavity (Fig. 6a). Moreover, NiCo NCs treatment accordingly caused a significant attenuation of the influx of neutrophils into the abdominal cavity (Fig. 6b and c), thereby demonstrating *in vivo* that NiCo NCs can effectively attenuate activation of inflammasomes in a peritoneal inflammation mouse model.

The dextran sodium sulfate (DSS) model is thought to usefully mimic the pathogenesis of human inflammatory bowel disease and has been extensively used to explore immune mechanisms of colitis [43], and there are reports that DSS-induced colitis in mice is mediated by NLRP3 inflammasomes [44–46]. To test the effect of NiCo NCs on DSS-induced colitis, DSS-treated mice were administered NiCo NCs via oral gavage. Upon induction of the DSS model, the control mice had more severe colitis than mice treated with NiCo NCs, as evidenced by a significant shortening of the colon (Fig. 6d and e) and an obvious loss of weight (Supplementary Fig. 12). Further supporting anti-inflammatory effects from NiCo NCs, H&E stained sections of colonic tissue sections showed that NiCo NCs treatment significantly reduced the damage of mucosal epithelium in a DSS mouse model (Fig. 6f). Linking our *in vivo* experimental results to our *in vitro* mechanistic studies, we found that the extent of IL-1β maturation was significantly attenuated by NiCo NCs treatment of DSS model mice (Fig. 6g). Thus, the observed capacity of NiCo NCs to relieve colitis symptoms relies, at least in part, on NiCo NCs’ capacity to reduce IL-1β maturation.

## CONCLUSION

Inflammasome activation is a major driver of many diseases, such as Alzheimer's disease, Parkinson's disease, pulmonary fibrosis, type II diabetes, gout and atherosclerosis. Macrophages serve as the primary defense mechanism of the body in the processing of nanoparticles, bringing about nanomaterials with a natural ability to target macrophages in comparison with small molecule drugs. This study provides an alternative approach to replacing small molecule drugs for the treatment of diseases mediated by hyperactivation of the inflammasome. In the present study, we found that NiCo NCs can effectively inhibit the activation of NLRP3, NLRC4 and AIM2 inflammasomes by blocking inflammasome assembly. We also show that NiCo NCs can inhibit neutrophil recruitment in an acute peritonitis mouse model, and can relieve symptoms in a colitis mouse model. Our work also establishes that these effects result from inhibition of IL-1β maturation. In sharp contrast to previous reports suggesting that inorganic nanoparticles typically induce inflammasome activation, we demonstrate that inorganic nanoparticles themselves can actually inhibit the activation of diverse types of inflammasomes. We have demonstrated that NiCo NCs exert broad-spectrum inhibitory effects on the activation of inflammasomes by downregulating *Neat1*. The mechanism by which NiCo NCs repress *Neat1* transcription also deserves further study. If so, then perhaps the commonly encountered, strong limitation of inflammasome activation that has to date faced engineered nanoparticle applications can be prevented or overcome by designing nickel- and/or cobalt-containing nanocrystals to achieve improved biocompatibility.

Additionally, it is crucial to understand how the physicochemical attributes of nanostructured materials, such as size, charge, morphology, composition and surface chemistry, affect immune responses by specific cell types. There is value in applying related knowledge to designing customized immunomodulatory nanomaterials for specific diseases. There is also a great need for studies that focus on addressing the long-term clinical safety of immunomodulatory nanomaterials, and rigorously optimizing them for their expected therapeutic effects before reliable clinical translation [47].

## METHODS

The details about the synthesis, characterizations, cell preparation, biological assay and analysis are in the Supplementary data.

## Supplementary Material

nwad179_Supplemental_File
