# Supplementary material for: Nickle-cobalt alloy nanocrystals inhibit activation of inflammasomes
Source: Natl Sci Rev. 2023 Jun 26;10(8):nwad179. doi: 10.1093/nsr/nwad179 (PMC10406336; doi:10.1093/nsr/nwad179)
Supplement: nwad179_Supplemental_File [file nwad179_supplemental_file.pdf]

Supplementary Information

Nickle-cobalt alloy nanocrystals inhibit activation of inflammasomes

Jun Lin[1,†], Liang Dong[1,4,†], Yi-Ming Liu[1,†], Yi Hu[1], Chen Jiang[1], Ke Liu[1], Liu Liu[1], Yong-Hong Song[2], Mei Sun[1], Xing-Cheng Xiang[5], Kun Qu[1,3,*], Yang Lu[2,*], Long-Ping Wen[1,*] and Shu-Hong Yu[1,*]

[1]Department of neurosurgery, The First Affiliated Hospital of USTC, School of Basic Medical Sciences, Division of Molecular Medicine, Department of Chemistry, Institute of Biomimetic Materials & Chemistry, Division of Nanomaterials & Chemistry, Hefei National Research Center for Physical Sciences at the Microscale, University of Science and Technology of China, Hefei 230027, China;

[2]Anhui Province Key Laboratory of Advanced Catalytic Materials and Reaction Engineering, School of Chemistry and Chemical Engineering, Hefei University of Technology, Hefei 230009, China;

[3]Institute of Artificial Intelligence, Hefei Comprehensive National Science Center, Hefei 230027, China;

[4]The Cancer Hospital of the University of Chinese Academy of Sciences (Zhejiang Cancer Hospital), Institute of Basic Medicine and Cancer (IBMC), Chinese Academy of Sciences, Hangzhou 310022, China;

[5]The WUT-AMU Franco-Chinese Institute, Wuhan University of Technology, Wuhan, 430070, China

***Corresponding authors.** E-mails: qukun@ustc.edu.cn; yanglu@hfut.edu.cn; lpwen@ustc.edu.cn; shyu@ustc.edu.cn

†Equally contributed to this work.

METHODS

Materials

Ni (II) acetylacetonate (Ni(acac)$_2$, 95%), Co(II) acetylacetonate (Co(acac)$_2$, 97%), SiO_2 nanopowder, adenosine triphosphate (ATP), monosodium urate crystals (MSU), poly(dA:dT), cytochalasin D, and FITC-dextran 70 KD were purchased from Sigma-Aldrich. Triethylene glycol (TREG), ethanol, polyvinylpyrrolidone (PVP, K30), nickel(II) chloride hexahydrate, cobalt(II) chloride hexahydrate and other chloride hexahydrate were purchased from the Shanghai Reagent Company. All reagents were purchased and used as received without further purification. Ultrapure LPS was purchased from Invitrogen. The antibody against mouse IL-1β was purchased from R&D. Anti-mouse caspase-1 (p20) and anti-ASC were purchased from Adipogen. Anti-human IL-1β was purchased from Sangon Biotech. Anti-human cleaved caspase1 was purchased from Cell Signaling Technology. FITC anti-mouse Ly6G, APC anti-mouse CD11B, anti-mouse CD16/32 was purchased from Biolegend.

Synthesis and characterization of NiCo alloy NCs, Ni NPs, and Co NPs

Ni(acac)$_2$ (0.0454 g), Co(acac)$_2$ (0.0184 g) and PVP (5 g) were mixed in TREG solution (30 mL). The mixture was heated at 120 ℃ on an oil bathing with magnetic stirring to form a transparent yellow solution. Then, the homogeneous solution was transferred into a microwave device (SINEO, MDS-6G). The temperature of solution was increased rapidly to 235 ℃ and held on at 235 ℃ for 15 min. After reaction finished, the formed jet-black solution was diluted with 30 mL ethanol. The product was centrifuged and washed with ethanol and water 2 times, respectively. For the synthesis of Ni NPs, Ni(acac)$_2$ (0.0649 g) was used in the synthesis process without the addition of Co(acac)$_2$, and the product was washed by ethanol and water 2 times, respectively. For the synthesis of Co NPs Co(acac)$_2$ (0.0613 g) was used in the synthesis process without the addition of Ni(acac)$_2$, and the product was washed by ethanol and water 2 times, respectively. TEM was performed on H-7650 (Hitachi, Japan). Scanning

transmission electron microscopy energy dispersive spectrometer (STEM EDS) element mapping was performed on JEOL-2010F. XRD pattern was obtained using a PANalytical X-Pert PRO MPD instrument (Netherland). Particle size and zeta potential were examined by a ZS-90 nanosizer (Malvern Instrument, UK). The content of Ni^{2+} and Co^{2+} ions were determined by inductively coupled plasma mass spectrometry (ICP-MS, Thermo fisher Scientific X Series 2).

Assessment of the zeta potentials of particle suspension

40 μg/mL NiCo NCs were incubated with three sets cell-free mediums in a humidified incubator at 37 ℃ for 30 min for subsequent zeta assessment. The three sets of cell-free mediums included (i) Opti-MEM, (ii) Opti-MEM + 10% FBS, (iii) Opti-MEM+10% FBS and then removed FBS. In (iii) group, NiCo NCs was washed with PBS three times and resuspended with fresh Opti-MEM to remove FBS [1].

Cell preparation and stimulation

Regents for cell culture were purchased from Gibco. Human THP-1 cells were differentiated by phorbol-12-myristate-13-acetate (PMA, 100 nM, Sigma-Aldrich) for 3 h. BMDMs were derived from tibia and femoral bone marrow cells which were cultured in DMEM contain 10% filtered L929 cell cultural supernatant. To test the effect of NiCo NCs on inflammasomes activation, opti-MEM supplemented with 100 ng/mL LPS was added. After priming by LPS for 3 h, NiCo NCs was added for another 2 h. Nigericin (5 mM for 45 min, Calbiochem), or ATP (1 mM for 45 min), or MSU (150 mg/ mL for 6 h), or SiO_2 (250 mg/ mL for 6 h) was added to stimulate the cells, respectively. In order to active AIM2 inflammasomes, 0.5 or 1 mg/ mL poly(dA:dT) was transfected. For salmonella infection to active NLRC4 inflammasomes, salmonella was precultured and BMDMs were infected in the next day for 1 h with 10 μL Salmonella culture (for 1 mL medium) and then incubated for another 5 h in the presence of gentamycin (Invitrogen).

Immunoblotting

To extract the protein in supernatant, 500 μL methanol and 125 μL of chloroform was added to 500 μL cell culture supernatant, followed by vortex and centrifugation. 500 μL methanol was added after discarding the upper phase. After centrifugation and removing the supernatant, the protein precipitate was dried for about 5 min at room temperature, which was further boiled in sample buffer for 10 min. For the immunoblotting assay, samples were separated by SDS-PAGE and were transferred onto polyvinylidene fluoride or nitrocellulose membranes, followed by the incubation with the primary and then the second antibodies and the visualization by an enhanced chemiluminescence kit.

ELISA

After indicated treatment, supernatants of cell culture, peritoneal perfusate and tissue culture were centrifuged to remove the precipitate, then the supernatants were assayed for the concentration of mouse IL-1β, mouse TNF-α (R&D) and mouse IL-18 (eBioscience).

LDH release assay

LDH release in the cell supernatant was quantified by employing the LDH activity kit (Beyotime, C0017) following the manufacturer's instructions. The relative LDH release was calculated as the percentage of LDH activity in the cultured cell supernatant (medium) relative to the total LDH activity in both the medium and the cells.

Immunofluorescence

After indicated treatment, BMDMs were fixed by paraformaldehyde (4%) and permeabilized with Triton X-100 (0.25%) for 5 min. BMDMs were blocked with 3% FBS after washing by PBS for 3 times. Then BMDMs were incubated with the primary antibody and then secondary antibody conjugated with Alexa543, which were observed by a Zeiss LSM800 confocal microscopy.

ASC SPECK assay

After removing the supernatants, BMDMs were washed by ice-cold PBS, and 500 μL of ice-cold buffer (20 mM HEPES-KOH, pH 7.5, 150 mM KCL, 1% NP-40, 1 mg/mL leupeptin, 0.1 mM PMSF, 1 mM sodium orthovanadate and 11.5 mg/mL aprotinin) was added [2]. Cell lysis was collected and 60 μL of lysate was took for immunoblotting. After centrifugation and discarding the supernatants, the obtained pellets were washed for 3 times and resuspended in PBS (500 μL). DSS (2 mM) was added and then incubated the mixture for 30 min with rotation at room temperature. Then, after centrifugation and removing the supernatant, the obtained cross-linked pellets were boiled for 5 min in 30 μL of sample buffer. Those samples were analyzed by immunoblotting

Transcriptome profiling (RNA-seq)

Total RNA of BMDMs were prepared using RNeasy Mini Kit (Qiagen, Hilden, Germany) according to the manufacturer's instructions. Then extracted RNA samples was analyzed by 2100 Bioanalyser (Agilent) and quantified using the ND-2000 (NanoDrop Technologies). Only RNA samples of high quality, with a RNA Integrity Number (RIN) of ≥7 and a quantity greater than 1.1 μg, were utilized for constructing the sequencing library. The next reverse transcription, library construction, and sequencing procedures were conducted as previously reported [3], following the manufacturer's instructions (Illumina), and these processes were carried out by Shanghai Mingcode Technology Co., Ltd.

Processing of RNA-seq data

Raw paired end reads were trimmed by Trimmomatic v0.38 [4]. The clean reads were mapped to the mouse genome (mm10) using HISAT2 v2.0.4 [5]. To obtain quantification scores for all mouse genes across all samples, raw counts were calculated using HTSeq v0.7.2 [6], and the raw counts matrix was shown in Table1. Gencode annotation vM25 was used in HTSeq. A cut-off value of 1 count was used as a detection limit across all samples. Raw gene counts were normalized by DEseq2 [7].

Differential expression gene analysis

Differential expression genes (DEGs) analysis across groups were performed by DESeq2 [7]. Significantly differentially expressed genes were filtered by foldchange and q-value in Fig. 4d and Supplementary Figs. 4 and 5. (Foldchange > 1.5, q-value < 0.1)

Real-time reverse transcription PCR (Real-time RT-PCR) analysis

Total RNA was isolated by RNeasy Mini Kit. (QIAGEN). cDNA was synthesized using a reverse transcription system kit according to the manufacturer's instructions (PrimeScript^{TM} RT reagent Kit with gDNA Eraser, RR047A, TaKaRa). Real-time PCR was performed using the KAPA SYBR FAST qPCR Kits (KK4601, Roche) following the manufacturer's protocol. Primer sequences for qRT-PCR are: Mouse *Neat1*_Forward, 5'-GGCACAAGTTTCACAGGCCTACATGGG-3'; Mouse *Neat1*_Reverse, 5'-GCCAGAGCTGTCCGCCCAGCGAAG-3'); Mouse *Gapdh*_Forward, 5'-GAGTATGTCGTGGAGTCTA-3'; Mouse *Gapdh*_Reverse, 5'-CTAAGCAGTTGGTGGTG-3'. Gene expression levels were normalized to *Gapdh* and analyzed using the comparative cycle threshold ($F = 2-\Delta\Delta Ct$) method.

ATAC-seq library construction for sequencing

ATAC-seq was performed as previously described [8]. Briefly, different treated BMDMs were washed with ice-cold 1× PBS twice, pelleted at 500×g for 5 min and resuspended in 50μl tagmentation mix (33 mM Tris-acetate, pH 7.8, 66 mM potassium acetate, 10 mM magnesium acetate, 16% dimethylformamide, 0.005% digitonin and 3.5 μl of Tn5 (TruePrep Tagment Enzyme, Vazyme, #S601-01)). The tagmentation reaction was carried out for 30 min at 37 ℃ and 500 r/min on a thermomixer (ALLSHENG MSC-100). The cells were washed with NIB buffer (10 mM Tris-HCl, pH 7.5, 10mM sodium chloride, 3mM magnesium chloride 0.005% digitonin) twice and pelleted down at 500×g, 4°C for 5 min. Cell were lysed with 14μl 10mM Tris-HCl, pH 7.5. 2μl lysis buffer (100mM Tris-HCl, pH 8.0, 0.4% SDS) and 0.2μl proteinase K for 15 min at 55°C and 500 r/min. The reaction

was then stopped with 4μl 10% TWEEN-20 and 0.4μl 100mM PMSF. Then, PCR were performed to amplify the library for 15 cycles with the following conditions: 72°C for 5 min, 98°C for 30 s, and thermocycling at 98°C for 15 s, 63°C for 30 s and 72°C for 5 min. After PCR amplification, the library was purified with SPRIselect beads (BECKMAN) at concentration of 0.5×-1.2× and MinElute Kit (QIAGEN).

Primary data processing and peak calling

The raw data from ATAC-seq was processed using the published ATAC-seq pipeline ATAC-pipe [9]. The '—MappingQC' module was used for read mapping and Bowtie was used to trim adapter sequences and map the reads to the mm10 reference genome. PCR duplicates were eliminated as described. The mapped reads were adjusted by +4/-5bp based on the read strand, ensuring that the first base of each mapped read corresponded to the Tn5 cleavage position. Subsequently, all mapped reads were extended to 50 bp, centered around the cleavage position. Reads aligned to repeated regions and chromosome M were excluded. The ATAC-pipe's '—PeakCalling' module was employed, with the options '—p1 3 --q1 5 --f1 1 -w 50' for peak calling using the MACS algorithm [10]. Peaks were then filtered and enriched regions were identified based on a posterior probability threshold of >0.99. Samples with the same treatment were grouped together for peak calling, and the resulting peaks from all categories were merged to create a unique peak list. Raw read counts corresponding to each peak in each sample were quantified and compiled into an $N \times M$ data matrix, where N represents the number of merged peaks, and M represents the number of samples. The matrix value $D_{i,j}$ denotes the raw read counts falling within peak i ($i = 1$ to N) of sample j ($j = 1$ to M). Subsequently, this data matrix was normalized using the 'normalize.quantiles' function from the 'preprocessCore' package in R and utilized for downstream analysis.

Assay for endocytosis of NiCo NCs into BMDMs

For ICP-MS assay, LPS primed BMDMs were treated by NiCo NCs (30 μg/mL) or cyto D (10 μM) + NiCo NCs (30 μg/mL) for 2h 45 min. The BMDMs were wash by PBS for 5 times to remove the nanoparticles on the cell surface, followed by Co quantitation with ICP-MS. For the FITC-dextran assay, LPS-primed BMDMs were treated with NiCo NCs (30 μg/mL), Cyto D (10 μM) or NiCo NCs +Cyto D for 2 h in the present of FITC-dextran (0.5 mg/mL). After washing twice with PBS, opti-MEM was added and the cellular fluorescence was examined by a Zeiss LSM800 confocal microscopy.

Animal experiment

The acute peritonitis animal model was performed on C57BL/6 mice (male, 6-8 weeks old, 20 g, Vital River Laboratory Animal Technology). In the acute peritonitis experiment, PBS, MSU (3 mg) and MSU (3 mg) + NiCo NCs (100 μg) were injected intraperitoneally. Mice were sacrificed 6 hours later, 5 mL PBS was injected intraperitoneally, then the level of IL-1β was measured by ELISA. And the recruitment of neutrophil (Ly-6G$^+$ and CD11b$^+$) into abdominal cavity was measure by Flow Cytometer, and anti-CD16/32 antibody served as block antibody. In the acute colitis experiment, the mice received oral administration of 2.5% DSS added drinking water for seven days. The mice in DSS+NiCo NCs group received a suspension of 5 mg/kg NiCo NCs in 100 μL PBS by intragastric administration at the day 2, 4 and 6. The blank group (without DSS in drinking water) received a same amount of PBS. The body weight of the mice was measured daily. All mice were sacrificed at day 8 for sample collection and the colons were measured to evaluate the severity of colitis. After rinsing in the PBS supplemented with 1% Penicillin-Streptomycin, two of five colons in each group were fixed by paraformaldehyde for subsequent H&E assay, and the rest colons were cultured in opti-MEM containing 1% Penicillin-Streptomycin for 12 h at 37 ℃. All the animal experiments were performed according to the protocols approved by the Animal Welfare Committee of University of Science and Technology of China.

Statistical analysis

All data were expressed as mean ± s.d. and analyzed by analysis of variance (ANOVA). *$P < 0.05$, **$P < 0.01$, and ***$P < 0.001$ were considered statistically significant.

REFERENCES

1. Zhang CC, Tang JB and Xie WJ *et al*. Mechanistic Observation of Interactions between Macrophages and Inorganic Particles with Different Densities. *Small* 2023; **19**: 2204781.
2. Coll RC, Robertson AAB and Chae JJ *et al*. A small-molecule inhibitor of the NLRP3 inflammasome for the treatment of inflammatory diseases. *Nat Med* 2015; **21**: 248-55.
3. Yu QN, Liu X and Fang JW *et al*. Dynamics and regulation of mitotic chromatin accessibility bookmarking at single-cell resolution. *Sci Adv* 2023; **9**: eadd2175.
4. Bolger AM, Lohse M and Usadel B. Trimmomatic: a flexible trimmer for Illumina sequence data. *Bioinformatics* 2014; **30**: 2114-20.
5. Kim D, Paggi JM and Park C *et al*. Graph-based genome alignment and genotyping with HISAT2 and HISAT-genotype. *Nat Biotechnol* 2019; **37**: 907-15.
6. Anders S, Pyl PT and Huber W. HTSeq-a Python framework to work with high-throughput sequencing data. *Bioinformatics* 2015; **31**: 166-9.
7. Love MI, Huber W and Anders S. Moderated estimation of fold change and dispersion for RNA-seq data with DESeq2. *Genome Biol* 2014; **15**: 550.
8. Chen X, Miragaia RJ and Natarajan KN *et al*. A rapid and robust method for single cell chromatin accessibility profiling. *Nat Commun* 2018; **9**: 5345.
9. Zuo ZQ, Jin YH and Zhang W *et al*. ATAC-pipe: general analysis of genome-wide chromatin accessibility. *Brief Bioinform* 2020; **20**: 1934-43.
10. Zhang Y, Liu T and Meyer CA *et al*. Model-based analysis of ChIP-Seq (MACS). *Genome Biol* 2008; **9**: R137.

Supplementary Figures

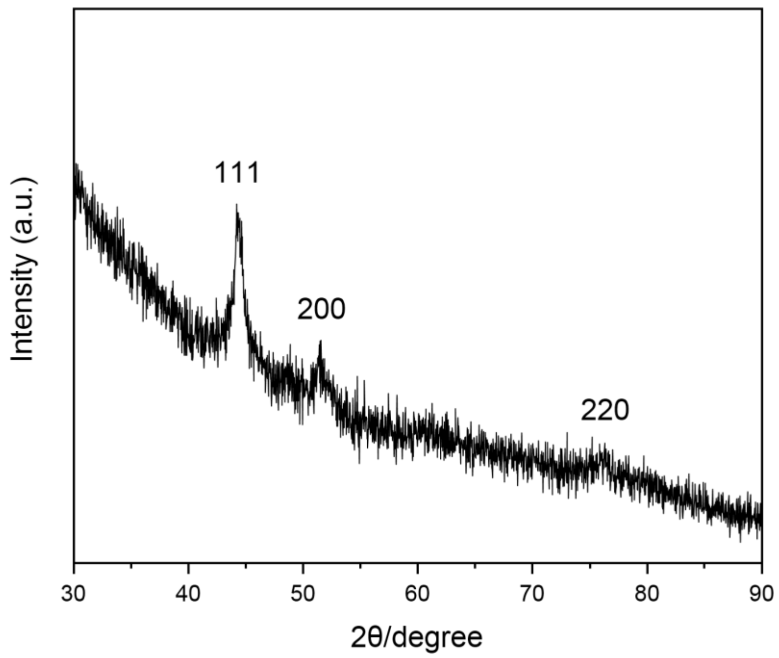

Supplementary Fig. 1. The XRD pattern of the as-prepared NiCo alloy nanocrystal.

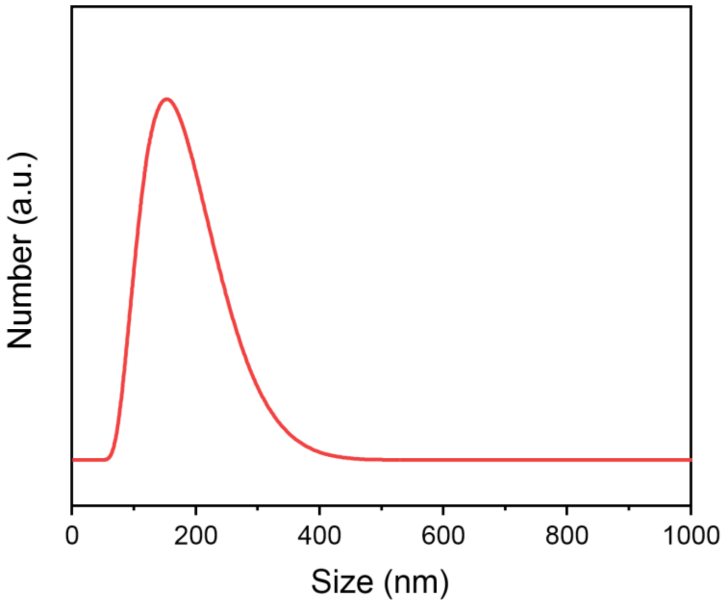

Supplementary Fig. 2. The DLS size distribution of NiCo NCs.

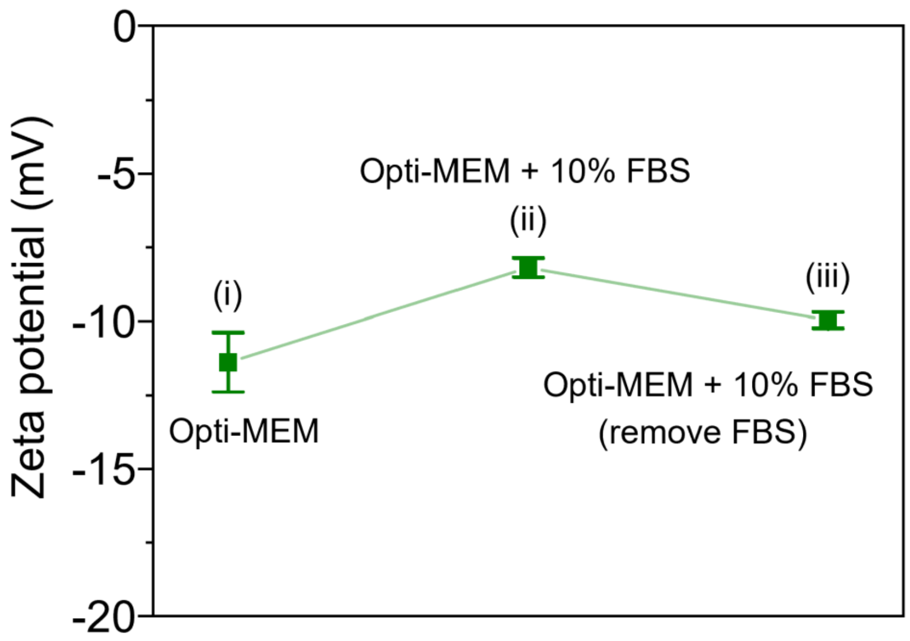

Supplementary Fig. 3. Zeta potentials of particle suspensions measured in three groups including (i) Opti-MEM, (ii) Opti-MEM + 10% FBS, (iii) Opti-MEM+10% FBS and then removed FBS. All data are presented as mean ± s.d., n = 3 independent experiments.

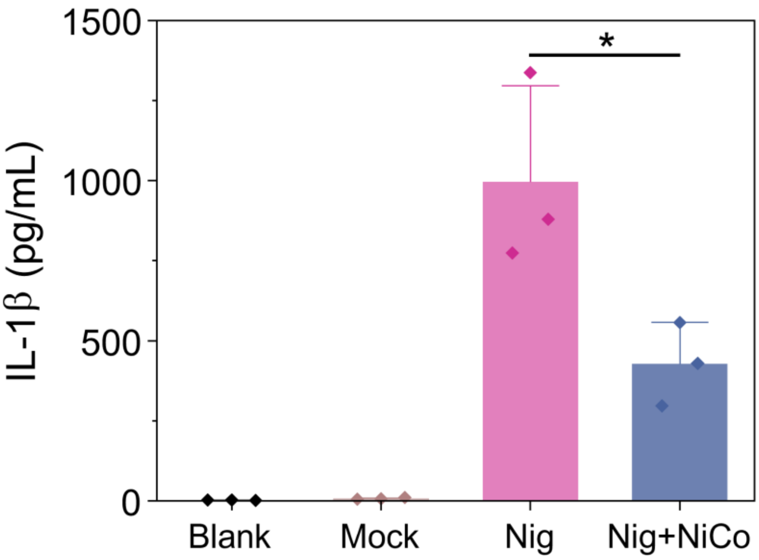

Supplementary Fig. 4. LPS-primed BMDMs were treated with nigericin for 40min or exposed to nigericin for 10 minutes followed by co-treatment with 30 μg/mL of NiCo NCs for an additional 30 minutes. Supernatants were analyzed by ELISA for the release of IL-1β. All data are presented as mean ± s.d., n = 3 independent experiments. Statistical significance was assessed using one-way analysis of variance (ANOVA). *$P < 0.05$.

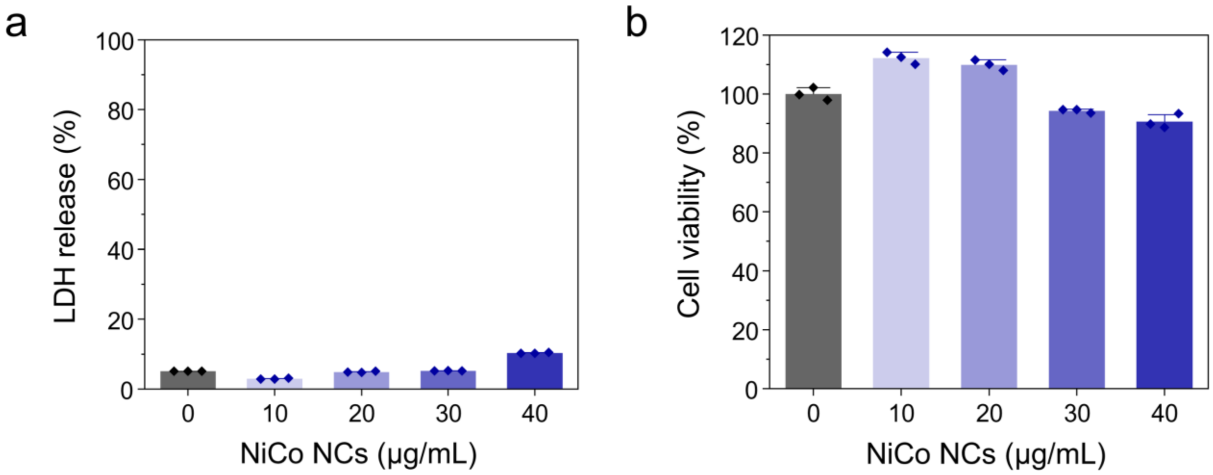

Supplementary Fig. 5 (a) LDH release assay in supernatants of BMDMs stimulated with different doses of NiCo NCs for 8 h. (b) The cell viability determined by MTT assay of BMDMs treated with different concentrations of NiCo NCs for 12 h. All data are presented as mean ± s.d., n = 3 independent experiments.

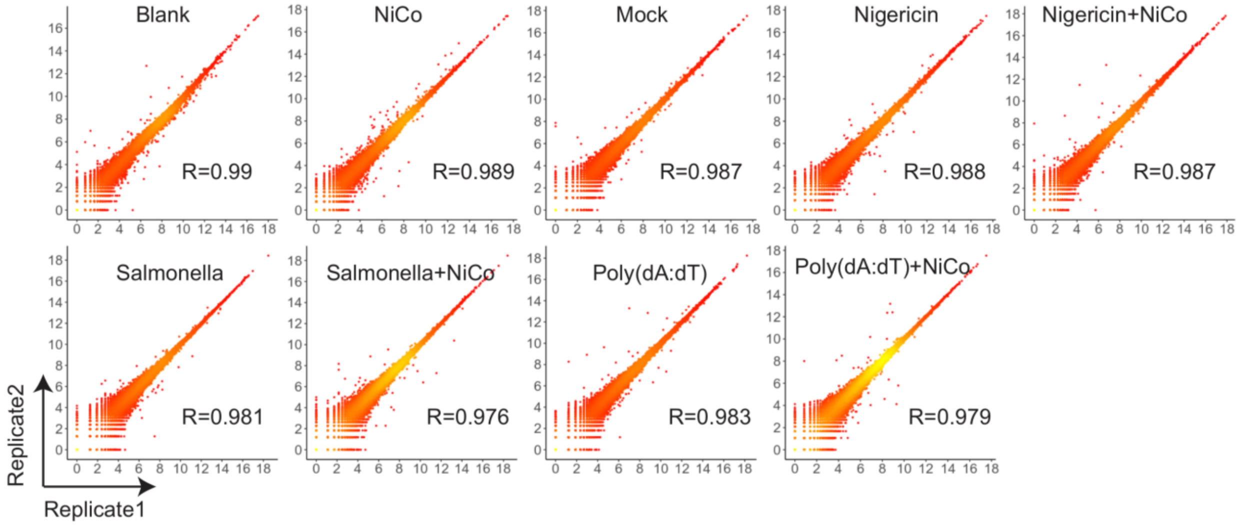

Supplementary Fig. 6. The scatter plot shows the Pearson correlation coefficient of RNA-seq signals between duplicate samples (18 samples under 9 conditions) in BMDMs. Blank = BMDMs without LPS-priming, Mock = LPS-primed BMDMs without stimulation.

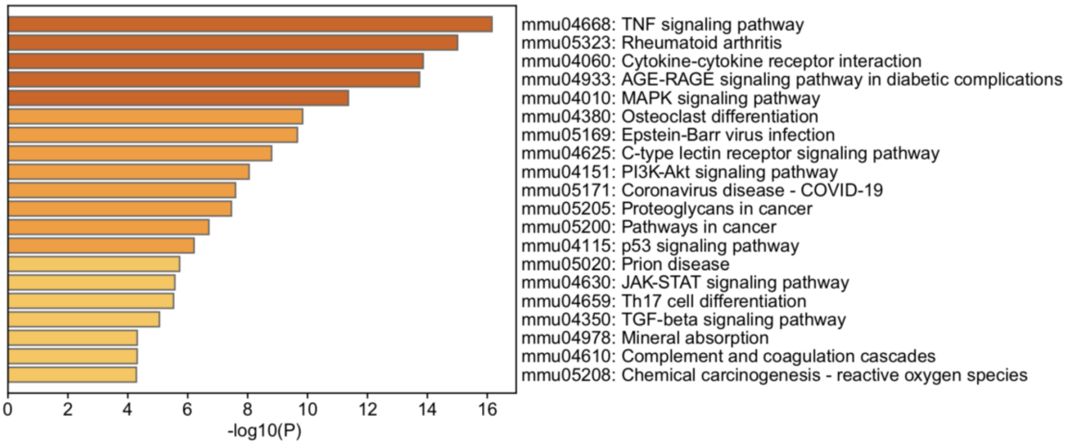

Supplementary Fig. 7. KEGG pathway enrichment analysis of differential expressed genes that upregulated by NiCo NCs treatment compare to Blank group.

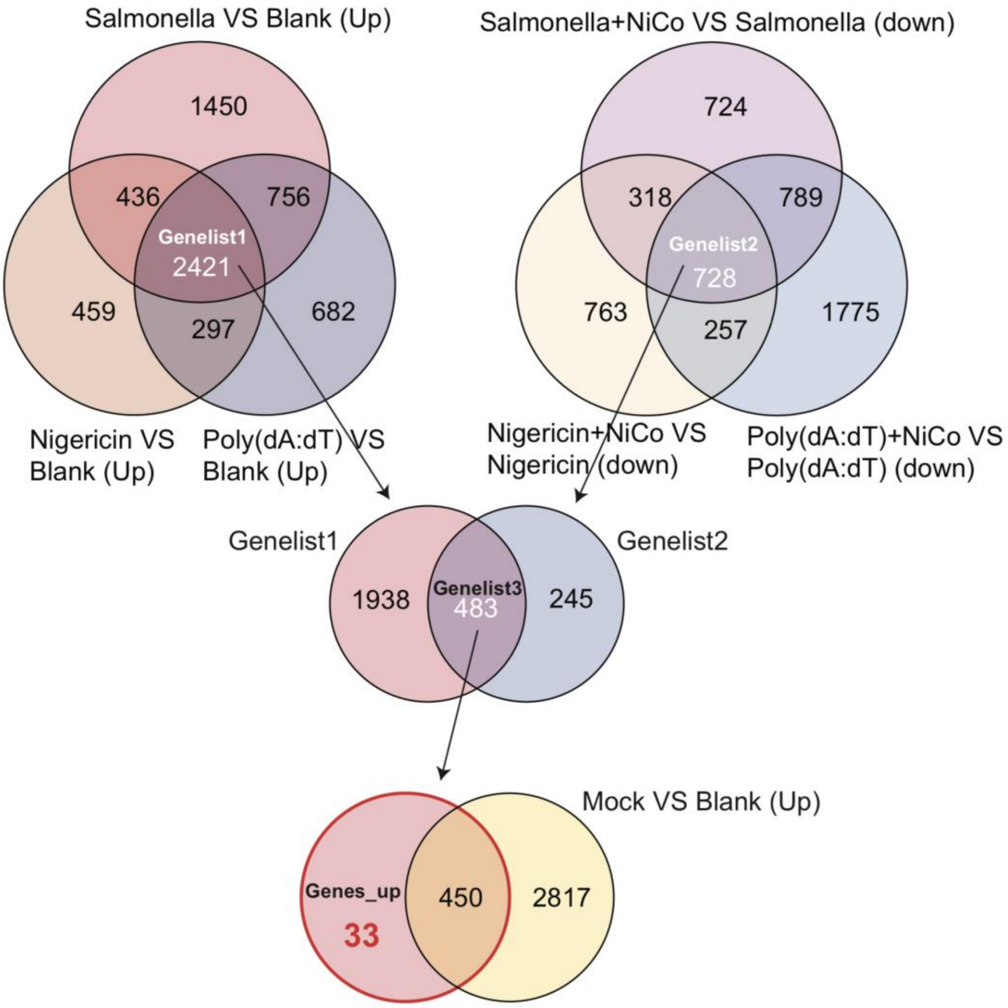

Supplementary Fig. 8. Workflow of identifying genes that are upregulated during inflammasome activation and are downregulated after NiCo NCs co-treatment (Genes_up in Fig. 4d).

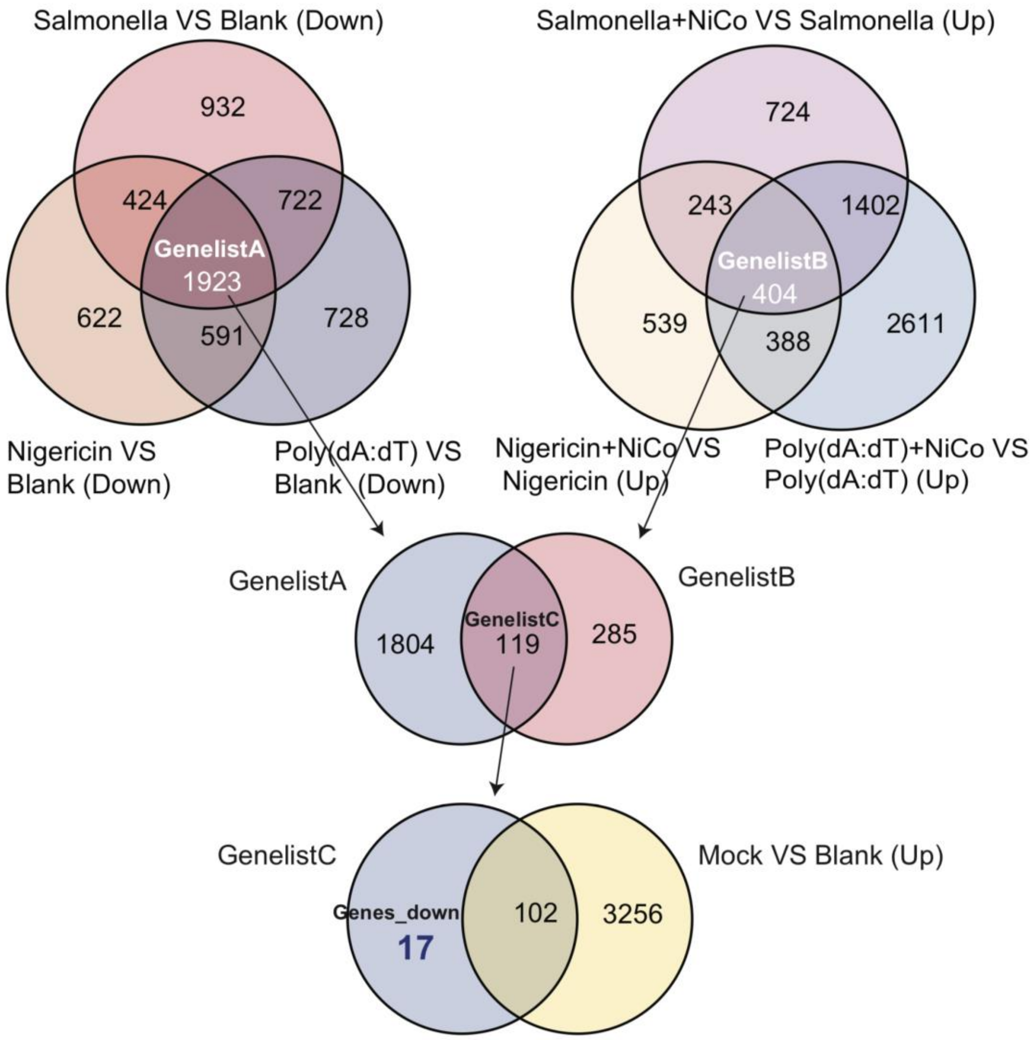

Supplementary Fig. 9. The way for identifying Genes_down. Workflow of identifying genes that are downregulated during inflammasome activation and are upregulated after NiCo NCs co-treatment (Genes_down in Fig. 4d).

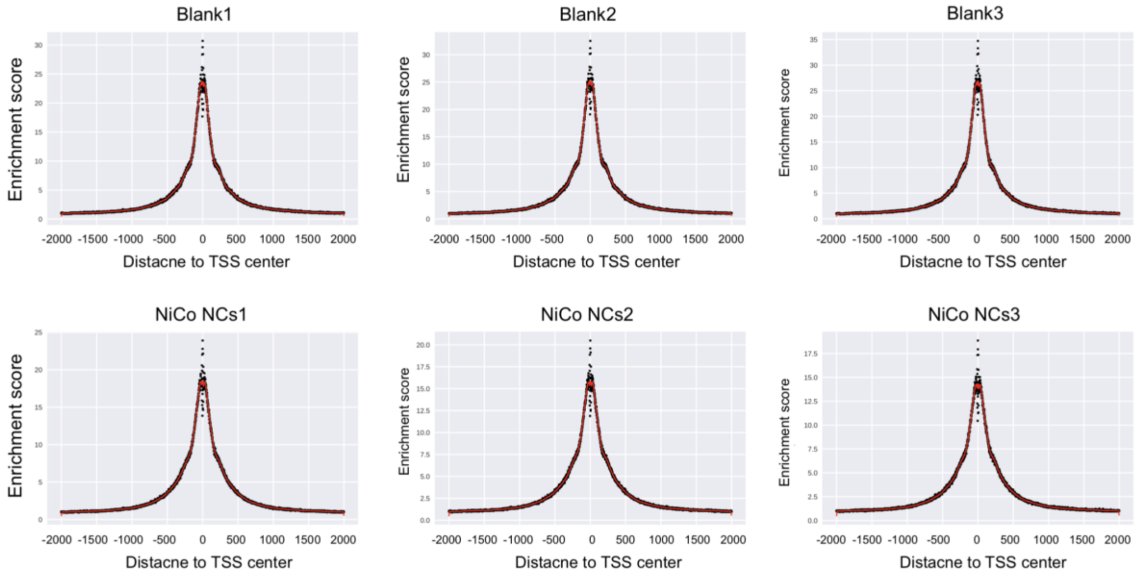

Supplementary Fig. 10. The transcription start site (TSS) enrichment scores of untreated (Blank) or NiCo NCs treated BMDMs.

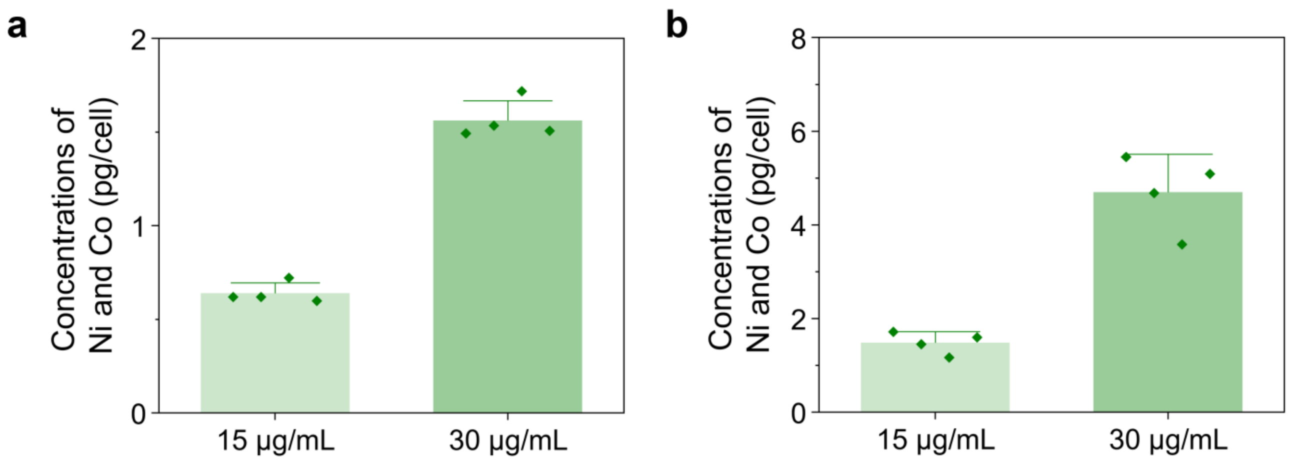

Supplementary Fig. 11. ICP-MS assay detection of Ni^{2+}+Co^{2+} concentration in (a) Raw264.7and (b) THP-1 cells treated with 15 μg/mL or 30 μg/mL NiCo NCs for 2 h after LPS priming. All data are presented as mean ± s.d., n = 4 independent experiments.

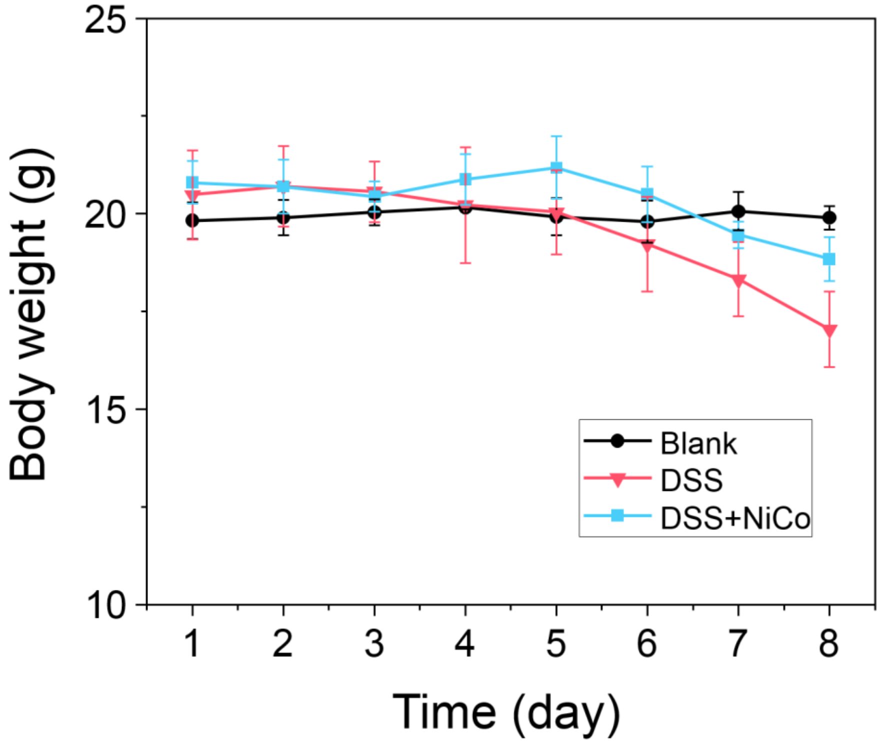

Supplementary Fig. 12. Body weight changes of C57BL/6 mice after treatment with different formulations. All data are presented as mean ± s.d., n = 5 independent experiments.
